# Coats disease in female population: A comparison of clinical presentation and outcomes

**DOI:** 10.3389/fmed.2022.879110

**Published:** 2022-08-04

**Authors:** Gwendoline Piquin, Thibaut Chapron, Youssef Abdelmassih, Gilles Martin, Catherine Edelson, Georges Caputo, Florence Metge

**Affiliations:** ^1^Pediatric Ophthalmology Department, Rothschild Foundation Hospital, Paris, France; ^2^Epidemiology and Statistics Research Center/CRESS, Institut National de la Santé et de la Recherche Médicale (INSERM), Institut National de la Recherche Agronomique (INRA), Université Paris Cité, Paris, France

**Keywords:** pediatric retinal disease, pediatric retinal detachment, Coats disease, bilateral disease, female population

## Abstract

**Purpose:**

To compare clinical characteristics at presentation and outcomes of Coats disease between females and males.

**Methods:**

In this retrospective, consecutive case series we included all children diagnosed with Coats disease in a single tertiary referral center. Initial clinical presentation, treatment and outcomes were collected.

**Results:**

A total of 158 children were included, of whom 29 (18.3%) were females and 11 (6.9%) had bilateral involvement. Age at diagnosis and disease stage were similar between females and males. Females had more bilateral involvement (*p* < 0.001) and tended to have a worse visual acuity at diagnosis (*p* = 0.05). At last follow-up, visual acuity and anatomical outcome after treatment were similar between genders.

**Conclusion:**

Female patients with Coats disease had more bilateral involvement and tended to have worse visual acuity at presentation. Clinical presentation and outcomes seemed to be similar between genders.

## Introduction

Coats disease is a rare, idiopathic, and non-hereditary retinal vascular disorder characterized by retinal telangiectasia, intraretinal and/or subretinal exudation without vitreoretinal traction. It was first described by Georges Coats in 1908 (1). The exact prevalence of this disease is not known but may vary among populations because of ethnic genetic susceptibility and/or exposition to environmental factors. A study carried out in the United Kingdom, estimated its incidence at 0.09/100.000 inhabitants (2). Coats disease is not associated with systemic manifestations, often occurs unilaterally, and is more frequent in males. It classically starts during the first or second decade with a natural evolution toward retinal exudation which can lead to a total exudative retinal detachment and neovascular glaucoma (3–5). The disease is staged according to Shields classification based on the presence and localization of vascular telangiectasia, exudation, exudative retinal detachment, and disease complications (3).

Although more frequent in males, Coats disease also affects females. Spitznas et al. (6) and Shields et al. (4) found a female prevalence of 28 and 24%, respectively (5). Few studies reported on Coats disease characteristics and outcomes in a female population: Shields et al. (5) reported a higher likelihood of advanced disease stages in females whereas Daruich et al. (7) reported similar disease presentation between females and males.

The purpose of our study is to compare the baseline characteristics and outcomes of Coats disease between females and males.

## Methods

This retrospective consecutive case series included all children diagnosed with Coats disease at the Rothschild Foundation Hospital, a tertiary referral center, between December 1998 and October 2019. This study was approved by the institutional review and local ethics committee and adhered to the tenets of the Declaration of Helsinki.

Coats disease was defined by the presence of retinal telangiectasia with intraretinal and/or sub-retinal exudation without vitreoretinal traction. Patients with uncertain or alternative diagnosis were excluded. Patients' demographics, clinical characteristics, imaging features, treatment modalities and outcomes after management of the disease were retrospectively collected.

Patients' demographics and presenting characteristics included age at diagnosis, gender, laterality of the disease and presenting sign [leukocoria, strabismus, reduced visual acuity (VA), incidental finding following screening]. Clinical characteristics at diagnosis included VA (Snellen converted to logMAR), intraocular pressure (mmHg) and anterior segment findings (iris neovascularization, cataract). Visual acuity was not available for preverbal children. All patients received a thorough fundus examination using either a slit lamp or indirect ophthalmoscopy as well as fundus imaging and fluorescein angiography (FA) with either a contact wide-angle fundus camera (RetCam, Clarity Medical Systems USA) or a non-contact ultra-widefield imaging (Optos PLC, Dunfermline, Scotland, UK) depending on the child's age. We analyzed the extent and localization of telangiectasia, exudation and ischemia, and presence and extent of retinal detachment. Staging of affected eyes was based on the Shields' classification (3). In brief, stage 1 is characterized by the presence of telangiectasia only, stage 2 by the presence of both telangiectasia and exudation (A: extrafoveal exudation, B: foveal exudation), stage 3 by the presence of an exudative retinal detachment (A1 subtotal extrafoveal detachment, A2 subtotal foveal detachment, B: total retinal detachment), stage 4 by the presence of a total retinal detachment with glaucoma, and stage 5 by the presence of end-stage changes: total retinal detachment, cataract, and phthisis bulbi. Contralateral eyes with anomalies such as peripheral vascular leakage, vascular tortuosity or peripheral non-perfusion were classified as “stage 0” since they didn't meet Shields' classification. Patients who were previously treated elsewhere were characterized as unknown for staging.

During examination, we paid particular attention to rule out pathologies that can mimic Coats disease such as retinoblastoma, retinopathy of prematurity (ROP), familial exudative retinopathy (FEVR), retinal capillary hemangioma, ocular toxocariasis and Coats-like syndromes combining bilateral exudative retinopathy with systemic conditions. Patients with bilateral disease underwent a brain MRI to exclude cerebro-retinal microangiopathy with calcifications and cysts (CRMCC) syndrome. When CRMCC was suspected on MRI, a genetic testing was performed.

Patients were divided into two groups according to gender. At presentation, functional and anatomical alteration was assessed. A severe functional alteration was defined by a VA less or equal to count fingers (CF) or a stage worse than 2A when the evaluation of VA was not possible. The presence of a retinal detachment defined a severe disease anatomically.

Treatment modalities included observation, laser photocoagulation, cryotherapy, intravitreal anti-vascular endothelial growth factor (anti-VEGF) injection, vitrectomy, and enucleation. At last follow-up both functional and anatomical outcomes were assessed. Some children were only referred to our institution for treatment and were therefore lost to follow-up since they went back to their referring center. Functional outcomes were evaluated based on VA at last follow-up. For anatomical outcomes, patients were classified in three categories: (1) no retinal detachment, (2) patients requiring vitrectomy, (3) anatomic failure with phthisis bulbi, total retinectomy, or enucleation.

All statistical analysis were performed using SAS 9.7 (SAS Institute Inc, Cary, NC). Descriptive statistics were reported as median and standard deviation (SD) for continuous variables, and as percentage for categorical variables. The categorical variables were tested for association with gender using Fisher exact test or Chi-square test. The continuous variables were compared between the two groups using Mann-Whitney U test. To assess the association between gender and Shields' classification, we divided the eyes into four groups as follows stage 1 and 2A (only telangiectasia or extrafoveal exudation), stage 2B (foveal exudation), stage 3A1 and 3A2 (subtotal retinal detachment), 3B and 4 (total retinal detachment) as not all stages were present in the two groups. Concerning VA, 4 groups were also created: Hand Moving (HM) or worse, CF, VA between 20/400-20/100 and VA better than 20/100. Significance was set at *p* < 0.05.

## Results

**Figure 1:**
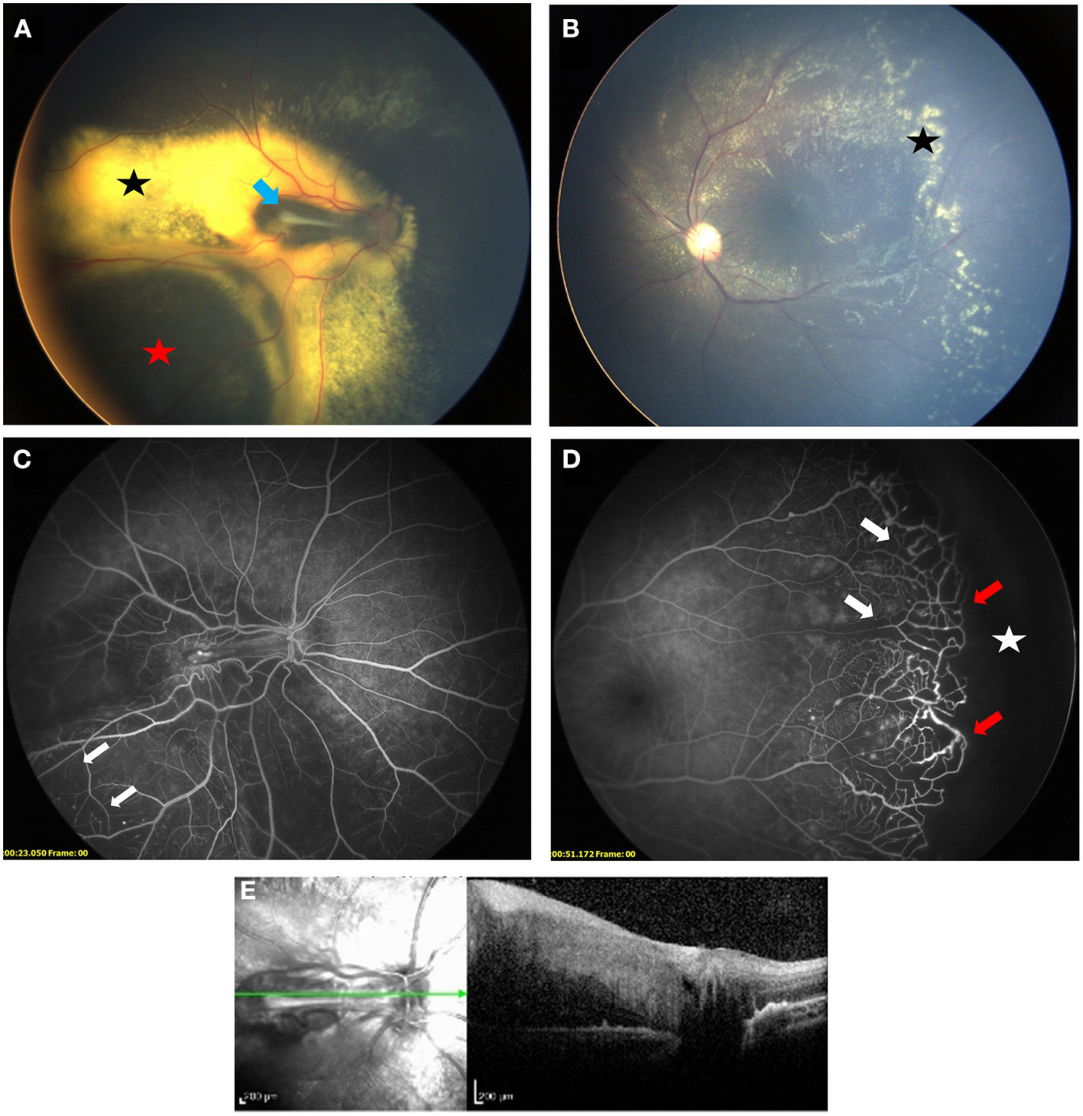
Bilateral Coats' disease in a 3-year-old girl. Right eye **(A)** is stage 3A1: fundus photograph shows an extrafoveal retinal detachment (red star) with exudates (black star) and a retinal fold at the level of the macula (blue arrow). Left eye **(B)** is stage 2A with extrafoveal exudation (black star). Fluorescein angiography shows capillary dropout (white arrows) in both eyes **(C)** right eye, **(D)** left eye) and a peripheral avascular retina (white star) lined with telangiectasia (red arrow) in the left eye. **(E)** SD-OCT section showing a thickening and hyperreflectivity of all retinal layers with preretinal fibrosis on top of the retinal fold.

**Figure 2:**
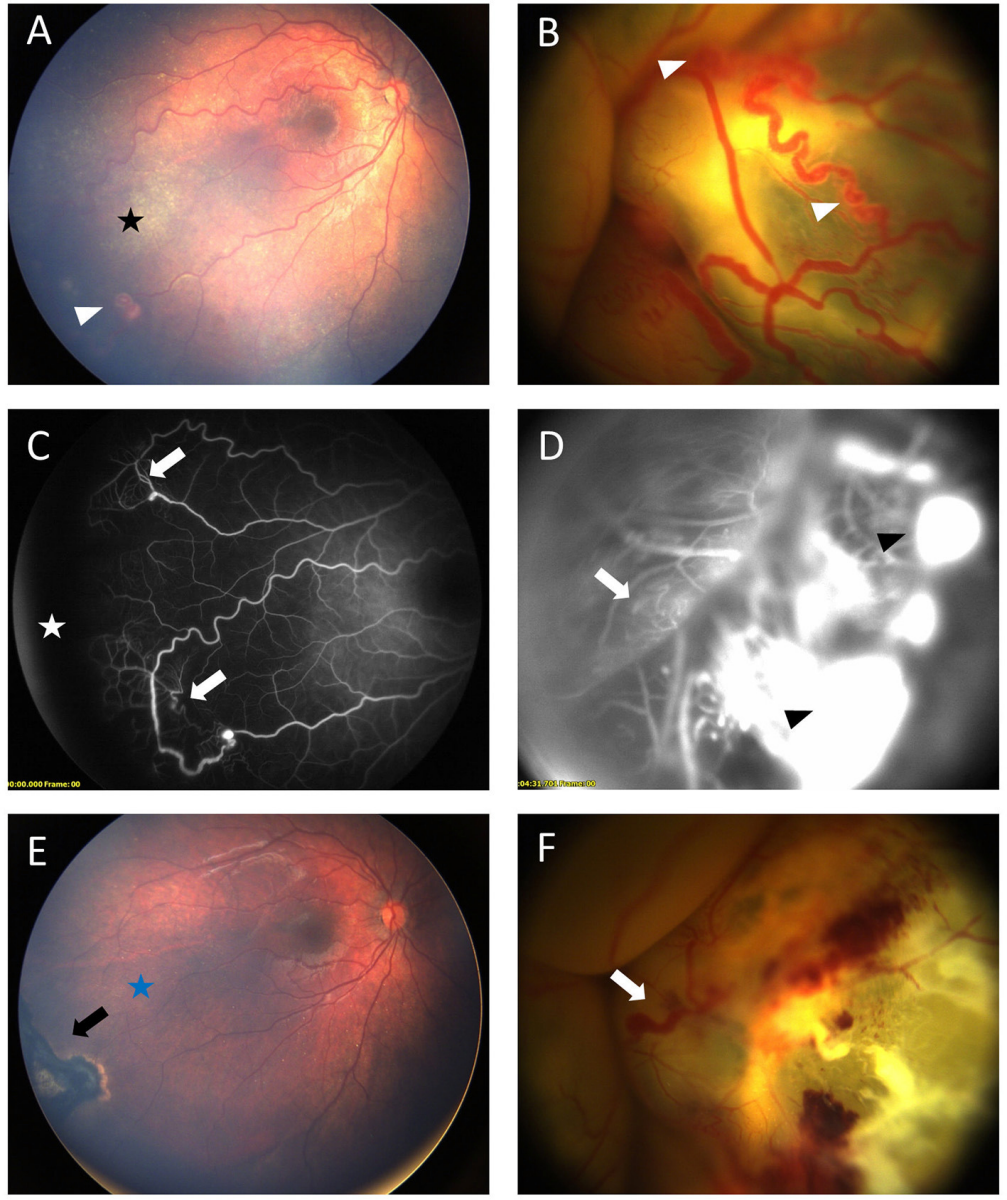
Bilateral Coats' disease in a 17-month-old girl. RetCam fundus photograph of the right eye **(A)** shows a stage 2A with moderate exudates (black star) sparing the fovea and ≪ light bulb ≫ aneurysms (white arrow head). Left eye **(B)** shows a stage 3B with a total retinal detachment covered with extensive aneurysms (white arrow head). Fluorescein angiography shows capillary dilatation and dropout (white arrows) on both eyes **(C)** right eye, **(D)** left eye and a peripheral avascular area (white star) on the right eye. Intense leakage is visible on the left eye (black arrow head). **(E,F)** shows fundus after first session of laser photocoagulation. On the right eye **(E)** black arrow shows retinal scar of photocoagulation and complete resolution of exudates (blue star). On the left eye **(F)** major exudates and dilated capillaries are still present (white arrow).

**Figure 3:**
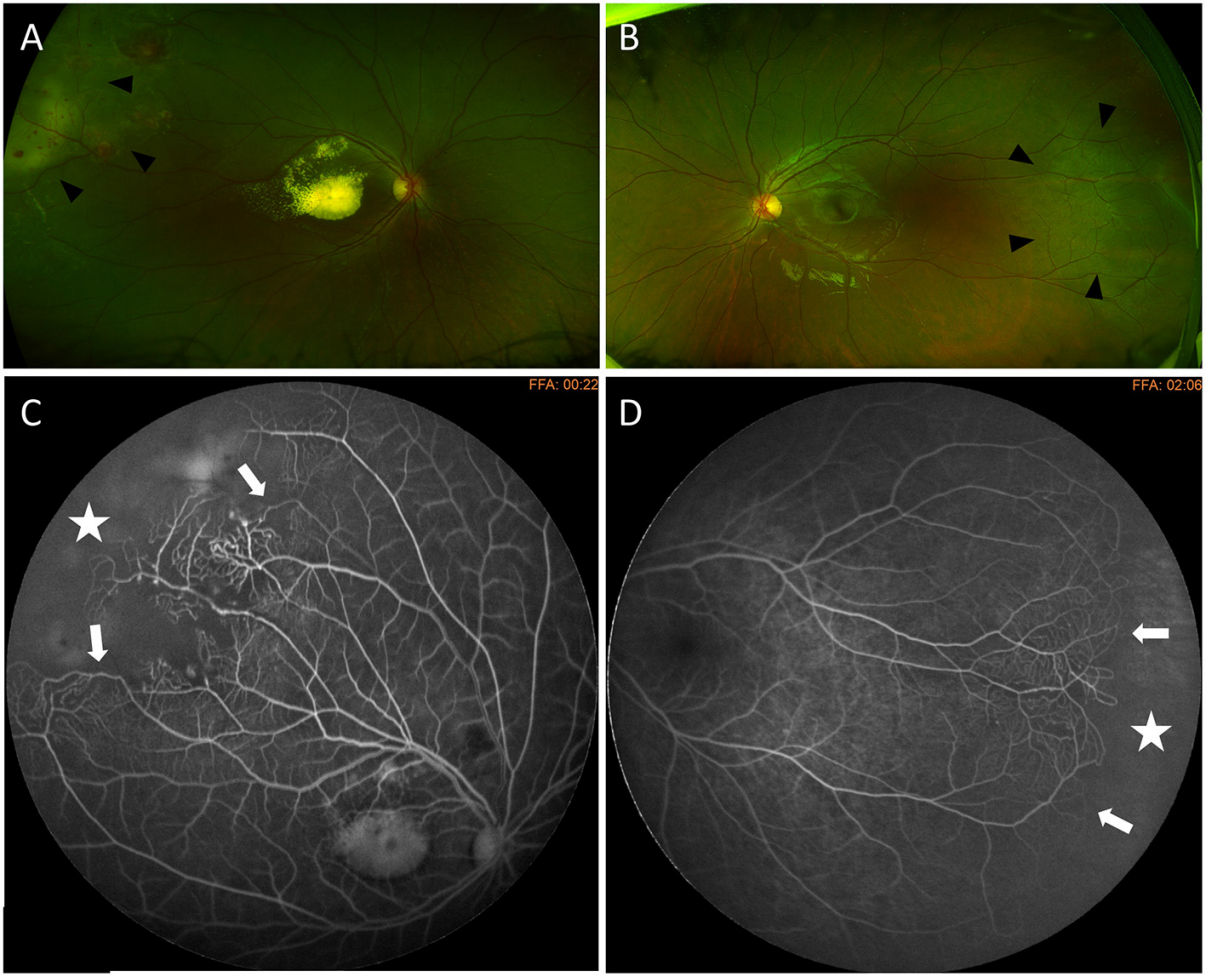
Bilateral Coats' disease in a 6-year-old girl. Optos wide-field fundus photography shows a stage 2B with macular exudation and peripheral telangiectasia (arrow heads) on the right eye **(A)** and telangiectasia located in the temporal periphery (arrow heads) on the left eye **(B)**. Fluorescein angiography shows capillary dilatation and dropout (white arrows) and a peripheral avascular area (white star) on both eyes **(C)** right eye **(D)** left eye.

**Figure 4:**
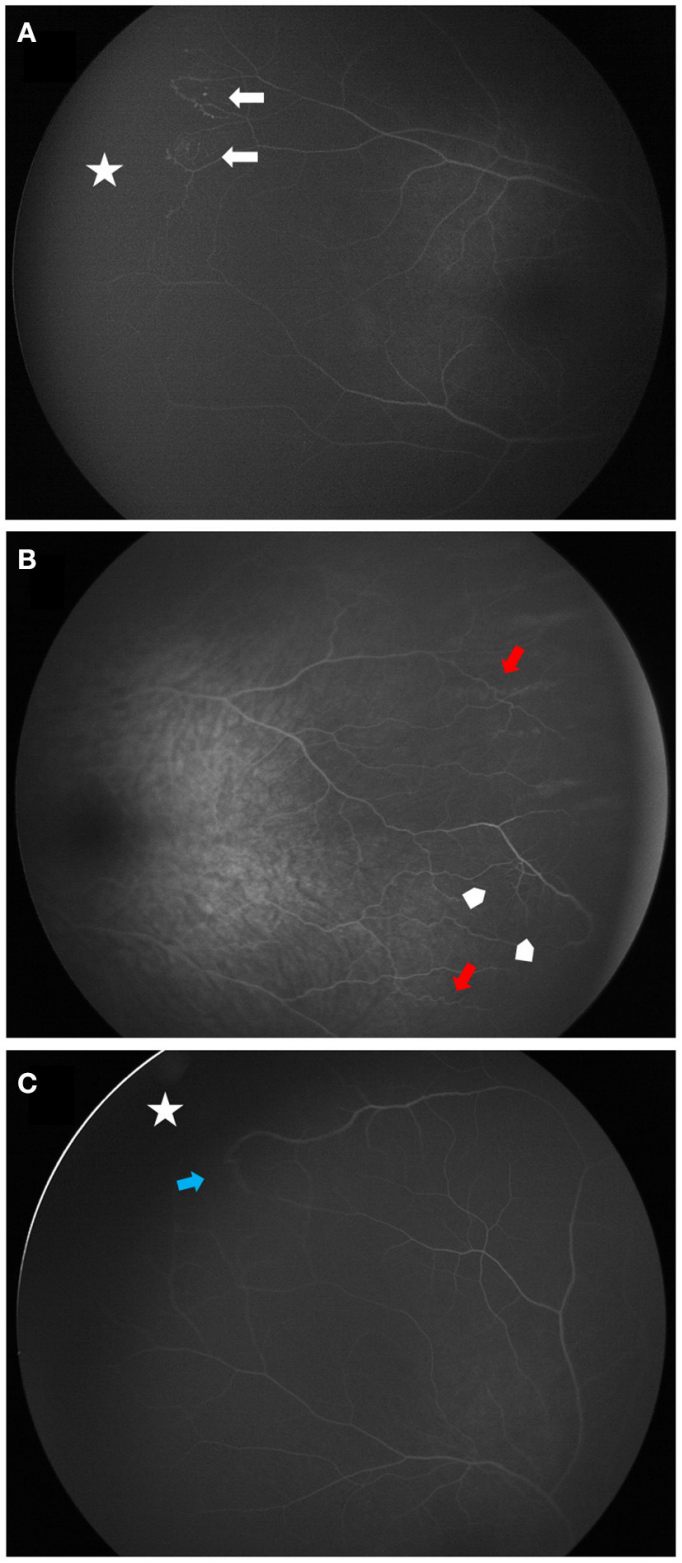
Wide angle fluorescein angiographies of contralateral vascular abnormalities in patients with Coats disease. **(A)** shows a peripheral avascular retina (white star) lined with telangiectasia (white arrows) (stage 1). **(B,C)** are classified stage 0. **(B)** demonstrates peripheral vascular tortuosity (red arrows) associated with dilatation of the capillary meshwork (arrow heads). **(C)** shows a peripheral avascular retina (white star) lined with a vascular anastomosis (blue arrow).

Out of the 161 patients (175 eyes) that met the clinical criteria for Coats disease, 3 (two females and one male) with a bilateral involvement were excluded because brain MRI found calcifications in favor of CRMCC syndrome, which was confirmed by the presence of a mutation on genes CTC1 or STN1. A total of 158 patients (169 eyes) were included of which 29 (18.3%) were females. Mean age at diagnosis was 7.1 +/- 6.4 years for females and 6.4 +/- 6.6 years for males (*p* = 0.60). No patient had a family history of Coats disease or other retinal vascular disease. All but 15 patients (two girls and 13 boys) received FA imaging. Children who did not received FA were not included in the bilateral involvement analysis. Bilateral disease was found in 11 (6.9%) patients and was more frequent in females (25.9 vs. 3.4%, *p* < 0.001). Bilateral involvement was clinically visible in five patients (Figures 1–3) while in the remaining six patients the involvement of the contralateral eye was only seen on FA (Figure 4). Visual acuity at diagnosis was available for 92 patients: 17.6% of girls vs. 21.3% of boys had a VA of HM or worse, 35.3% of girls vs. 10.7% of boys could count fingers, 29.4% of girls vs. 28% of boys had a VA between 20 and 400 and 20/100 and 17.6% of girls vs. 40% of boys had a VA better than 20/100. Visual acuity tended to be worse in females (*p* = 0.05). Presenting signs and symptoms were: leukocoria for 20.8% of girls vs. 19.8% of boys, strabismus for 25% of girls vs. 25.6% of boys, visual impairment for 37.5% of girls vs. 26.7% of boys, and 16.7% of girls vs. 27.9% of boys were asymptomatic. Presenting signs and symptoms were comparable between groups (*p* = 0.63). Stage of the disease at diagnosis was available for all but one girl who was initially seen and treated elsewhere. The diagnosis of Coats was never made at stage 1 for females but in 13 patients (10.1%) for males. None of the males and three females (10.7%) had a stage 4 disease. No patient had a stage 5. Baseline characteristics are shown in Table 1.

**Table 1:** Comparative analysis of baseline characteristics between males and females.

	**Females** **N** = **29**		**Males** **N** **=** **129**		
					**p**
**Mean age at diagnosis** in years ± SD (*N* = 155)		7.1 ± 6.3		6.4 ± 6.6	0.60
	***n***	**%**	***n***	**%**	***p***
**Initial BCVA (*****N*** **=** **92)**					0.05
≤ HM	3	(17.6)	16	(21.3)	
CF	6	(35.3)	8	(10.7)	
20/400–20/100	5	(29.4)	21	(28.0)	
>20/100	3	(17.6)	30	(40.0)	
**Presenting symptom or sign (*****N*** **=** **110)**					0.63
Leukocoria	5	(20.8)	17	(19.8)	
Strabismus	6	(25.0)	22	(25.6)	
Vision impairment	9	(37.5)	23	(26.7)	
Asymptomatic (routine examination)	4	(16.7)	24	(27.9)	
**Clinical staging based on Shields' classification (*****N*** **=** **157)**					
1 Telangiectasia only	0	.	13	(10.1)	
2A Telangiectasia and extrafoveal exudation	3	(10.7)	14	(10.9)	
2B Telangiectasia and foveal exudation	13	(46.4)	45	(34.9)	
3A1 Extrafoveal subtotal retinal detachment	5	(17.8)	24	(18.6)	
3A2 Foveal subtotal retinal detachment	0	.	5	(3.4)	
3B Total retinal detachment	4	(14.3)	28	(21.7)	
4 Total retinal detachment and neovascular glaucoma	3	(10.7)	0	.	
**Bilateral involvement (*****N*** **=** **143**)					<0.001
No	20	(74.1)	112	(96.6)	
Yes	7	(25.9)	4	(3.4)	
**Clinical bilateral involvement (*****N*** **=** **143**)					0.004
No	23	(85.1)	115	(99.1)	
Yes	4	(14.8)	1	(0.9)	

*BCVA, best-corrected visual acuity; CF, count fingers; HM: hand motion; N, number of patients and SD, standard deviation*.

Disease classification was combined into four groups, stages 1 and 2A, stage 2B, stage 3A, and stages 3B and 4. At presentation, classification, functional and anatomical alterations were comparable between groups (*p* = 0.48, 0.21 and 0.89, respectively, see Table 2).

**Table 2:** Comparative analysis of composite criteria at baseline between males and females.

	**Females** **N** = **29**		**Males** **N** **=** **129**		
	***n***	**%**	***n***	**%**	***p***
**Initial presentation (*****N*** **=** **157)**					0.48
Telangiectasia ± extrafoveal exudation (stage 1 + 2A)	3	(11.1)	27	(20.9)	
Foveal exudation (stage 2B)	13	(46.4)	45	(34.9)	
Subtotal exudative retinal detachment (stage 3A1 + 3A2)	5	(17.9)	29	(22.5)	
Total exudative retinal detachment (stage 3B + 4)	7	(25.0)	28	(21.7)	
**Functional severity (*****N*** **=** **157)**					0.21
Yes	25	(89.3)	102	(79.1)	
No	3	(10.7)	27	(20.9)	
**Anatomical severity (N=157)**					0.89
Yes	12	(42.9)	57	(44.2)	
No	16	(57.1)	72	(55.8)	

*Functional severity was defined as best-corrected visual acuity ≤ counting fingers or a disease stage worse than 2A and anatomical severity was defined by the presence of retinal detachment. N, number of patients*.

In the 11 patients with a bilateral form, the disease was always asymmetric. The least affected eye was classified as stage ≪0≫ in 6 patients, stage 1 in 2 patients, stage 2A in two patients, and stage 2B in one patient. The 6 eyes with stage ≪0≫ had anomalies only visible on FA: peripheral vascular leakage (two patients), vascular tortuosity (four patients) and peripheral non-perfusion associated with capillary meshwork abnormalities (dilatation and/or rarefaction) (two patients). Six of these 11 patients received laser photocoagulation on the least affected eye to treat telangiectasia in five eyes, and to treat a large peripheral avascular zone in one eye classified as stage 0. Characteristics of patients with bilateral disease are shown in Table 3.

**Table 3:** Clinical characteristics of patients with bilateral disease.

**Patient N°**	**Gender**	**Age at presentation (years)**	**Presenting sign**	**Stage**		**Initial BCVA**		**Treatment**		**Final BCVA**	
				**RE**	**LE**	**RE**	**LE**	**RE**	**LE**	**RE**	**LE**
1	Female	6	Routine examination	2A	0	20/20	20/20	Laser	Observation	20/20	20/20
2	Male	1	Strabismus	2B	3A1	NA	NA	Laser	Laser	NA	NA
3	Female	3	Strabismus	3A1	2A	HM	20/32	Laser	Laser	NA	NA
4	Female	26	Floaters	0	2A	20/20	20/20	Observation	Laser	20/16	20/16
5	Male	4	NA	3A1	0	20/200	20/20	Laser	Observation	CF	20/20
6	Female	9	Strabismus	1	4	20/40	NLP	Laser	Antivegf cyclodiode	20/16	NLP
7	Female	1	Leukocoria	2A	3B	NA	NA	Laser	Vitrectomy	20/20	NLP
8	Male	NA	NA	1	0	NA	NA	Laser	Laser	20/320	20/40
9	Male	8	Routine examination	0	1	NA	NA	Observation	Laser	NA	NA
10	Female	7	Visual impairment	0	2B	20/20	CF	Observation	Laser	20/20	20/250
11	Female	6	Routine examination	2B	1	20/100	20/20	Laser	Laser	20/100	20/20

*BCVA, best-corrected visual acuity; LE, left eye; NA, not available; RE, right eye*.

Functional and anatomical outcomes at last follow-up were similar between both groups (*p* = 0.41 and 0.82, respectively). Twenty-one (72.4%) females and 98 (77.8%) males had an attached retina without surgery, 6 (20.7%) females and 21 (16.7%) male required vitrectomy, and 2 (6.9%) females and 7 (5.6%) males required total retinectomy, enucleation or had phthisis bulbi. Results after management are shown in Table 4.

**Table 4:** Comparative analysis of outcomes between males and females.

	**Females** **N** = **29**		**Males** **N** **=** **129**		
	***n***	**%**	***n***	**%**	***p***
**Final BCVA** **(*****N*** **=** **128)**					0.41
≤ HM	13	(48.1)	36	(35.6)	
CF	2	(7.4)	15	(14.9)	
20/400–20/100	7	(25.9)	21	(20.8)	
>20/100	5	(18.5)	29	(28.7)	
**Anatomical outcome (*****N*** **=** **155)**					0.82
No retinal detachment	21	(72.4)	98	(77.8)	
Patient requiring vitrectomy	6	(20.7)	21	(16.7)	
phthisis bulbi or total retinectomy or enucleation	2	(6.9)	7	(5.6)	

*BCVA, best-corrected visual acuity; CF, counting fingers; CI, confidence interval; HM, hand motion; N, number of patients*.

## Discussion

In this retrospective study, we reviewed all patients with Coats disease and compared the characteristics and outcomes between genders. Females represented 18.3% of patients in our study. Shields et al. and Spitznas et al. reported similar frequencies ranging from 16 to 28% (3–6, 8), while Daruich et al. found slightly lower frequencies of 14 and 11% (7, 9, 10).

Despite being usually a unilateral disease, 11 patients (6.9%) had bilateral involvement in our study. The rate of bilateral involvement showed a lot of discrepancies in literature (4–6, 8, 11–14). Some of these studies were based solely on fundus photographs (4), while others used FA imaging (6). The use of FA and more recently wide-field imaging systems helped detect more peripheral abnormalities (11–13). The improvement in detection of peripheral vascular anomalies using wide-field imaging systems may improve the detection and the classification of Coats disease, as it has already been demonstrated in other vascular pathologies such as diabetes (15). Jung et al. showed that 8.9% of patients with clinically unilateral disease had vascular abnormalities on the fellow eye revealed by FA whereas Rabiolo et al. found a higher rate of 77.8% (12, 13). Brockmann et al. (14) reported on the presence of vascular changes on ultra wide-field FA in all the fellow eyes (100%) of middle-aged patients (mean age of 33.2 years and range between 12 and 66 years) with Coats disease and concluded that Coats disease seems to be an asymmetric bilateral disease, with abnormalities predominant in one eye. Vascular abnormalities consisted of peripheral non-perfusion, leakage, vascular tortuosity and occlusion or dilatation of capillaries (11, 12, 16–18). Among the 11 patients with bilateral involvement in our study, 6 had a normal contralateral fundus and abnormalities were only seen on FA. Since the definition of Coats disease is based on the presence of telangiectasia visible on indirect ophthalmoscopy or fundus photographs and since these angiographic abnormalities do not fit the Shields' classification, we classified these findings as “stage 0.”

When the disease is bilateral, it is important to consider other etiologies that can mimic Coats disease such as ROP, FEVR, and systemic diseases that can be associated with Coats-like retinopathy such as facioscapulohumeral muscular dystrophy, aplastic anemia and CRMCC. However, in these cases the involvement is more symmetrical. It is therefore important to perform a brain MRI to look for cysts and calcifications of the brain in patients with bilateral involvement especially when anomalies are clinically visible in both eyes. A total of eight patients had a bilateral involvement that was clinically visible with no evidence of systemic manifestations on clinical examination. After brain MRI, cerebral calcifications were found in three of the eight patients who underwent a genetic testing confirming CRMCC syndrome, and were excluded. The remaining five patients had an unremarkable brain MRI and therefore genetic testing was not indicated.

Little is known on initial presentation and outcomes of Coats' disease in females. In a recent study, Daruich and Munier compared the clinical presentation of Coats disease between males and females and found no differences between these two groups in terms of age at diagnosis, presenting signs and symptoms, and severity at diagnosis based on Shields' classification (7).Shields et al. reported a higher likelihood of advanced disease stages in females (5). Several studies found that a younger age at diagnosis is associated with a more advanced stage of the disease and a poorer visual prognosis (4, 10, 19). In our study, females had similar age at diagnosis, presenting symptoms, stage group and VA. However, there was a tendency toward worse visual acuity in female (*p* = 0.05) without reaching statistical significance, which can be due to the limited number of patients.

Furthermore, we found that bilateral involvement was more frequent in females. Eleven patients had bilateral involvement (seven females, 64%) of which 5 had clinically visible anomalies (four females, 80%) while the rest were only diagnosed on FA. All eyes with clinically visible anomalies underwent laser while only one eye with stage 0 was treated for a large peripheral avascular zone. To our knowledge, no other study has investigated this association. In their series of 112 patients, Spitznas et al. (6) did not test the association between bilaterality and gender but found, in the age population of 0–20 years, that 17.6% (three out of 17) of females and 2.4% of males had bilateral involvement, which is similar to our study. It seems that in the first two decades, females have more bilateral involvement than males. One hypothesis is the presence since birth of bilateral lesions more frequently in the female population. Another hypothesis is males might develop bilateral involvement later in the course of the disease.

The limitation of the present study is that it is retrospective and that data such as VA was not always available for young children. We believe that the limited number of patients especially in the female group and the lack of VA available at presentation might have limited the statistical power of VA analysis between genders. Furthermore, since our institution is a tertiary referral center, severe and atypical cases maybe overrepresented because of referral bias. Finally, the use of wide-field FA increased the sensitivity of detecting peripheral abnormalities that may have been previously missed in other series.

In conclusion, 18.3% of patients with Coats' disease were females and bilateral involvement (overall 6.9%) was more frequent in females (25.9 vs. 3.4%, *p* < 0.001). Fluorescein angiography can be useful to evaluate contralateral involvement and brain MRI should be performed for all patients with bilateral involvement. Age at presentation, stage groups and VA was similar between genders, with a tendency toward a worse VA at presentation in the female group. It seems that Coats' disease could present or become bilateral more frequently or earlier in female population. Outcomes after treatment were comparable between genders. Larger studies should be done to better compare VA at presentation between genders and to compare outcomes according to proposed treatments.

## Data availability statement

The raw data supporting the conclusions of this article will be made available by the authors, without undue reservation.

## Author contributions

GP, TC, and FM contributed to conception and design of the study. GP and TC organized the database. TC performed the statistical analysis. GP wrote the first draft of the manuscript. GP, TC, and YA wrote sections of the manuscript. All authors contributed to manuscript revision, read, and approved the submitted version.

## Conflict of interest

The authors declare that the research was conducted in the absence of any commercial or financial relationships that could be construed as a potential conflict of interest.

## Publisher's note

All claims expressed in this article are solely those of the authors and do not necessarily represent those of their affiliated organizations, or those of the publisher, the editors and the reviewers. Any product that may be evaluated in this article, or claim that may be made by its manufacturer, is not guaranteed or endorsed by the publisher.
